# Erosive Wear Mechanisms of Materials—A Review of Understanding and Progresses

**DOI:** 10.3390/ma18071615

**Published:** 2025-04-02

**Authors:** Tong Deng

**Affiliations:** The Wolfson Centre for Bulk Solids Handling Technology, Faculty of Engineering and Science, University of Greenwich, Central Avenue, Chatham ME4 4TB, UK; t.deng@greenwich.ac.uk

**Keywords:** erosive wear mechanisms, impact dynamics, material properties, erosion resistance, aggressiveness of solid particles

## Abstract

Erosive wear of materials caused by solid particles leads to severe damage on the surface of structure materials and results in mechanical failures. Erosion has been extensively studied for many years in terms of mechanisms, material properties, and impact dynamics. Since the early 21st century, little progress has been made on the evaluation of surface erosive failure due to multiple impacts of particulate solids. The major difficulty is the enormous number of variables involved in the erosion process. However, the existing theories are only able to take a few of them and end up with many assumptions on the others. In summary, the influential factors on erosion can be classified as impact dynamics (such as velocity and angles), mechanisms of material failures (deformation, cutting, and cracking), and material properties of solids and the surface (hardness, toughness, ductility, and brittleness). In this paper, erosion mechanisms and progress from the existing theories have been reviewed critically, which gives a better understanding of the phenomenon. Based on the review of the influential factors in terms of contributions to the process, proper evaluation methods of the erosion process have been discussed, which leads to further thinking of better assessments.

## 1. Introduction

Erosive wear caused by solid particles is a common phenomenon and is defined as a volume of material removed by a rigid body. Material removals lead to functional failures of the materials and alternatively result in systematic failure of the equipment, e.g., punctures in pneumatic conveying pipes. Erosive wear is quite popular on many occasions such as in turbines in power generation, aeroplanes, high-speed trains, and material handling in industrial processes. Because of the severe damage caused, erosion has been studied extensively since the 1950s. In 1958, Finnie [1] developed a theory of erosion based on cutting failure and Bitter [2,3] improved the theory by considering both deformation and cutting failures. The influences of the material properties and the impact dynamics on erosion have been investigated throughout the 1960s to 1980s [4]. These studies considered the influences of the impact by a single particle such as velocity; angle; mechanisms of material failures, e.g., fatigue and cracking; material properties including particle size and shape, hardness, and strain rates; and the process such as work hardening and thermal effects. However, because the number of influential factors studied increased significantly, the studies faced incredible challenges in selecting suitable parameters for the reliable prediction of erosion, especially for multiple-particle impacts. Consequently, numerical simulations caught more attention on tracking historical changes in erosion to have a better prediction since the late 20th century [5] but little progress has been made on better assessment of erosion [6]. Despite the studies, the investigation of erosion still suffered from a lack of understanding of the failures in the erosion process and better assessment methods.

Erosion by single-particle impacts is the simplest case, in which the damage on both particles and the surface is visible and measurable. In most cases, the surface material is more important than the solids as the erosion would result in functional failure of the equipment. Unlike the solids, the surface material suffers from accumulated damage from multiple impacts and a progressive failure in erosion. In the early stage, theoretical studies particularly focused on erosive failure mechanisms due to a single impact and tried to correlate the failure with the material properties [5,6]. However, with more influences considered, the theories still had huge assumptions in the calculations.

The erosion theories were summarised in 1995 and it was concluded that there was no single predictive equation or a group of limited equations that can be good for practical uses [7]. In the work, a list of parameters selected in erosion models was presented, which included the material properties of erodent particles and surface and impact dynamics. The constants used in the equations were from one up to ten. In the equations that have been reviewed, the largest number of variables used in a single equation was 26, and the least number was two. As erosion could be due to multiple collisions, a single or a group of dynamic equations cannot describe the process precisely and predict the process of multiple-particle impacts. With computing technology developed, numerical simulations of erosion become the majority such as using finite element methods (FEMs) [8,9,10,11]. On the other hand, discrete element methods (DEMs) also show advantages in tracking multiple impacts with historical changes in material properties in the contacts [12,13]. However, although these numerical models can provide better predictions on erosive wear, they still face barriers on how to deal with the huge variables involved in calculations and the numerous assumptions of the erosion mechanisms [14,15,16].

Nowadays, preventing erosive damage or improving the wear resistance of materials still demands a better understanding of erosive wear mechanisms. The existing theories of erosion face great challenges in practical analysis, particularly for the historical progression of erosion in the analysis. Although a number of reviews on erosion theories [6,7], assessments [16], and applications [17,18] have been conducted, the challenges of dealing with enormous uncontrollable variables in the analysis have not been addressed and solutions of better erosion assessments or improving numerical simulations incorporating evolving material properties are limitedly presented. In the paper, erosion mechanisms and some important influential factors are discussed in terms of their contributions to the erosion process, which could give a better understanding of erosive failure. Thinking of erosive wear more fundamentally may simplify the assessments of erosion, which could help the development of standardised erosion testing methods to ensure comparability across the studies.

## 2. Erosion Mechanisms

Erosive wear of material is complicated and is defined as material removals by solid particle impacts resulting in functional failures of the material [19]. The erosive mechanisms can be a number of reasons including plastic deformation, cutting, fatigues, brittle fracture, melting and tribo-chemistry, etc. [16,20,21,22]. Different materials behave differently in erosion processes such as metals, ceramics, elastoplastic materials, and structural composites [8,17]. Even the materials in the same category can have different erosive behaviours because of the progressive changes in material properties during the impacts.

### 2.1. Fundamentals of Erosive Wear

**Figure 1:**
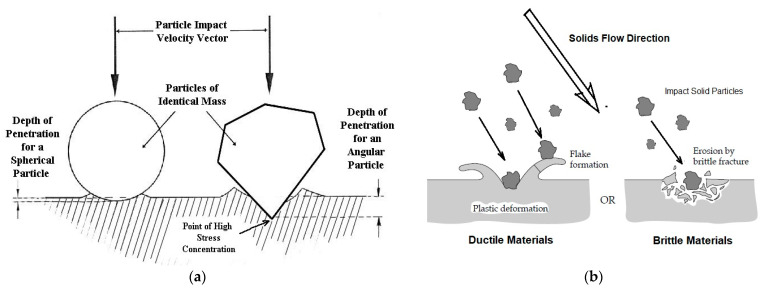
Surface material under impacts: (**a**) normal impact; (**b**) oblique impact.

Erosion of material is recognised as a progressive process by multiple particle impacts, but traditional studies are used to undertake single particle impact tests for mechanism study and use material removals for quantifying erosion failure. The failure depends on how severe damage is caused by the accumulation of the material removals. Without considering the damage of solid particles, erosion of surface material can be simply described as a progressive volumetric loss of the surface material under impacts (see Figure 1). In most studies, the responses of surface materials under impacts were focused on including the material properties and changes in the material properties, although particle dynamics was also considered in the analysis [1,2,3,7].

Single-particle impact seems a bit simple. However, the process is still quite complicated and the influential factors can be from ten to a hundred different characteristics regarding material properties such as hardness and toughness [7]. On the other hand, more variables of solid particles need to be considered including particle size, shape, and density. However, erosion is a dynamic progressive failure of materials, which is subject to the impacts of solids, the compressive pressure generated at contacts, etc. [18]. Therefore, in terms of failure mechanisms, the removal of surface materials will be due to the loss of bonding strength between the parts and the separated parts removed from the surface in multiple stages by further failures in the material.

### 2.2. Elastic and Plastic Deformations

The deformation of material under compression was thought as the first stage of material failure in erosion [19]. Erosion can happen in three stages: material deformed, failures in structures, and material removal. As the process may reach different stages during a single impact, visible damage caused by the impact appears as a result of material removal. Alternatively, material removals can be caused by multiple plastic deformations under repeated dynamic loadings such as fatigue for metals and fractures for brittle materials. Erosion needs to be treated as an accumulation of continuous material failures and consequent material removal as a result.

The concept of elastic–plastic deformation was introduced by Bitter [2,3] in his extensive study of Finnie’s work on cutting theory where Finnie faced difficulty at a normal impact angle [1]. Bitter successfully incorporated the deformation theory based on Hertz’s contact theory [23]. By the deformation-failure mechanism, erosion was nicely described in terms of impact angles. Although afterwards this theory has been used in most of the theoretical analysis of erosion, the prediction could not always match or proportion to the erosion measured in many practical applications [24], which revealed that there must be other mechanisms involved in erosion. Nevertheless, the deformation theory became very popular in the analysis of erosion failure mechanisms.

Material deformation can be classified into two types: elastic and plastic deformation. An elastic deformation in material science is defined as a temporary change in material dimension where the dimension reforms to the original point after the deformation is released [25]. For erosion, whatever the impact velocity is, elastic deformation always happens but it may not lead to any material failure or removal. With Hertz’s theory [23], elastic deformation can be calculated with material properties such as Young’s modulus and yield strength. If erosion is treated as a quasi-static loading, it can be described as the absorption of a definite amount of kinetic energy [26], but this is not always the truth. Impact is an impulse loading and involves energy transmission (stress waves) through the body during the impact [27]. The difficulty of high stress loading was considered in the calculation of surface yielding and maximum shear stress for many different materials [28,29] because erosion behaves like a high-stain rate loading process. In most cases, erosion is caused by multiple impacts, which leads to either multi-cycles deformation or linear load deformation, failure can happen at any stage if the load or the accumulated load is beyond the critical value [19,30].

### 2.3. Material Removal by Cutting

Cutting is a special plastic deformation, which can be treated as a failure mechanism, especially for ductile or elastomeric materials [31]. Finnie used ‘cuttings’ from the impact area for ductile material erosion [1] and Bitter added the yielded deformation for normal impacts [2,3]. Following that, Hutchings [4] used the concepts of ‘cutting’ or ‘ploughing’ for several erosion mechanisms claimed, e.g., fragment of ‘tips’, ‘piling-up’ of thin layers [32,33], and adiabatic shear failure [34], etc. Especially for high angles of impingement, mechanisms of plastic deformation leading to failure become more complicated [35,36]. Nevertheless, since the 1970s, progress based on plastic deformation and cutting has been significant [7,24,37]. For ductile materials, hardening and fatigue theories were used for analytical models [38,39]. For brittle materials, the fracture theory in cuttings was used in simulations [40,41].

With the studies, it can be realised that cutting is only a failure form of plastic deformations and leads to a large material removal commonly. Cutting is a dynamic plastic deformation in two dimensions which involves vertical and horizontal plastic deformation. Vertical plastic deformation may not contribute to material removal directly, but it creates changes in material properties in erosion such as hardening, which can cause further material removal. Both are important to material removal if erosion happens due to the cutting mechanism.

### 2.4. Fatigue and Fracture Failures

Fatigue and fracture of materials under high-cycle compression in the contact region are thought to be the main wear mechanisms along with shearing, displacement of materials, or material melting happened at the same time [42]. Fatigue is a gradual damage or a change in material properties of a material structure or a component subjected to cyclic loads, eventually leading to a complete failure of the structure [43]. Repeated plastic deformations can lead to material removal under multiple solid impacts. Initial plastic deformations may not cause a structural failure but it creates a change in material properties during the erosion process. Remarkably, the loading stress level causing fatigue failure is much lower than the maximum allowable stresses for a single, static applied load, which frequently happens in erosion [44]. Fatigue failure typically develops in three stages: crack initiation, crack propagation, and termination. Crack is initiated by solid particle impacts on the locations with elevated material stress. The size of cracks can be tiny, not more than 0.5 mm frequently [45]. The crack propagates with repeated impacts. Once the crack crosses with other cracks, the part completely ruptures from the material as a failure. Erosion due to fatigue failures can happen to any material including elastomers [46], which has been studied since the 1960s and included in many theories [47,48]. Nowadays, the challenges in erosion analysis by fatigue mechanisms remain the same as the other erosion mechanisms, which involve many uncontrolled variables [17,49].

**Figure 2:**
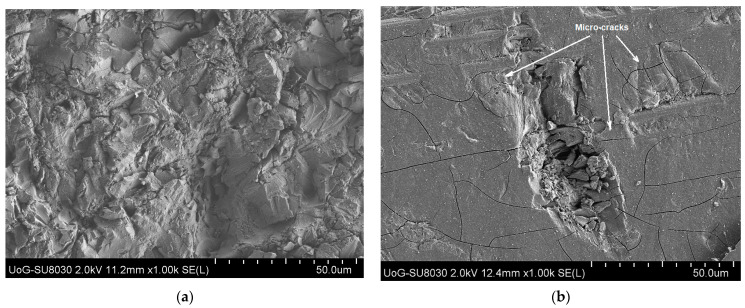
Image of erosion failures [52]: (**a**) fatigue fracture surface, neat epoxy impacted at 90°; (**b**) microcracks, CFR epoxy composite impacted at 30°.

Fracture failure is the forced separation of a material into two or more parts. The fracture of a brittle material can happen without any appreciable plastic deformation (i.e., energy absorption), but ductile fracture involves large plastic deformation before separation [41,49]. Fracture failure in erosion is caused by the loading stress exceeding the material’s inherent strength, and a part of the surface material is removed from the surface by the fluid stream [50,51]. The facture failure mechanism happens to any crystal materials such as polymers and polymer composites as shown in Figure 2.

### 2.5. Erosive Failures of Materials

Erosive wear mechanisms have been studied for many years and reviewed in many studies [6,7,16,17,53]. In summary, it was concluded that plastic deformation (or plastic flow) [54,55]; abrasive cutting (or ploughing) [56]; fatigue of ductile materials [4]; fracture of brittle materials [57]; and localised melting [22,58] were the main failures. Some uncommon failures were also mentioned previously such as crystal lattice degradation [59] and corrosive–erosive wear [60,61]. Some important failure mechanisms are discussed here in detail including plastic deformation, cutting, fatigue cracking, and fracture failure. Some less popular ones are not such as localised melting [4]. However, the localised heat generated in erosion is not a direct failure mechanism unless the heat is high enough to melt the material [58,62].

Although the failure mechanisms of erosion have been studied for many years [63], it is still confused with different materials or different scenarios. Any erosive failure of materials always begins with a plastic deformation whether it is small or not, and then the deformed parts are broken into parts as the failure completes and removes the parts as follows. Therefore, erosion failure is a progressive failure by plastic deformation, fatigue cracking, and fracture cracking, although localised heat has an influence [6].

It is thought that erosion is caused by a combination of the failure mechanisms [4] and the influential factors are not independent, i.e., any deformation will subject to impact dynamics, material properties of the solids and the surface, and the alternative changes in the material properties due to previous impacts. The factors analysed are increased rapidly when the theories are developed, and it ends up at 180 s in more than 30 s analytical equations [7]. A universal theory of erosion seems impossible. Using a single equation or a group of equations to predict erosion is never achieved.

## 3. Solid Impact Dynamics

Erosion involves solid impact dynamics where solids strike a surface at a velocity and remove some of the materials from the surface. Finnie [1] studied the erosion of ductile materials by a single rigid abrasive grain under Newton’s law. Afterwards, erosion was generally described in terms of particle velocity at different impact angles. However, the erosion rate is hard to link with the impact dynamics directly.

In 1972, Finnie [64] summarised particle dynamics including particle velocity, impact angle, localised contact, momentum, and rotation, although some of the factors discussed were not dynamic such as particle size, shape, and concentrations. Since that time, solid impact dynamics have become fundamentals in all erosion analyses [4,7]. Currently, solid impact dynamics is used in explanations of erosion mechanisms, but the challenge of using that for the theoretical analysis of erosion is the uncontrollable variables such as particle sizes and shapes. All these variables are interlinked in the process. The concept of kinetic energy carried by solid particles was used to reduce the number of variables involved in the analysis, but it still faces challenges from size variations and different energy transmissions during the impact [6,24]. From this point, solid impact dynamics may need to be considered separately in the erosion assessment from the other factors such as material properties so the evaluation can be simplified.

### 3.1. Kinetic Energy of Solids and Impact Velocity

The kinetic energy of projectile solids has been used for characterising the impact damage of surface materials in many applications [6]. During the impact, the kinetic energy of solids converts into several other types of energy, and it is believed that the material failure is caused by the energy of plastic work in the target material. However, it is hard to calculate the energy proportion responsible for the size of the crater, but the amount of the energy can be in proportion to the total kinetic energy of the solids carried as shown in Equation (1).

(1) $$E_{k}=\frac{1}{2}m_{p}v_{p}^{2}=\frac{1}{2}\rho_{p}V_{p}v_{p}^{2}$$

where *E_k_* is the initial kinetic energy, *m_p_* and *v_p_* are the mass and velocity of the particle, *ρ_p_* is the density of the solids, and *V_p_* is the volume of the solids which is subject to the size of particles.

It is realised that the particle impact velocity is one of the important dominant factors influencing the erosion of materials as the erosion increases with an increased kinetic energy of impact particles. At a higher impact velocity, even particles with smaller sizes may produce significant erosion as reported in the literature [65,66]. The exponent of the impact velocity in Equation (2) varies in a range of 2.5–4 for ductile materials [39], 2.7–5 for brittle materials [40], and 1.5–3.5 for plastics and elastomers [16]. Although the values outside the ranges were also reported [67], the functional dependence of erosion rate on particle impact velocity follows a power law that has been widely used to correlate the erosion rate to the particle impact velocity as follows:

(2) $$Erosion\;rate\;\left(\varepsilon \right)\propto E_{k}\propto \;A_{R}{\left(v_{p}\right)}^{n}$$

where the exponent ‘*n*’ is a power index of the velocity and the *A_R_* is a constant which depends on the mechanical characteristics of the material and the particles and can be a function of impact dynamics such as impact velocity and impact angle. It has been observed that the velocity exponent is mostly greater than two for most materials [5]. So, any small variation in its value can significantly affect the accuracy of the erosion rate prediction. However, the focus on the accurate determination of velocity exponent value for the correct prediction of erosion generates great challenges in the estimation of the influences from other effects such as the energy transmissions.

### 3.2. Energy Transmission and Impact Angle

The kinetic energy carried by the solids converts into several forms of other energies after the impact including the energy that turns into elastic or plastic work in the particle or the target material, the energy of heat generated, and the kinetic energy remaining in the bouncing particle [68]. The energy resulting from plastic deformation or localised melting may cause material failures due to the impact and alternative removals, but this is not always the truth. In energy transmissions, the material removals caused by plastic deformation only happen when cracking is established. In an oblique impact (Figure 1b), it shows that the impact has two vectors: a horizontal component and a vertical component. In this case, the vertical component of an impact leads to a deformed region in the material or microcracks if the material is brittle. The plastic deformation in the horizontal direction is likely to remove the deformed material easily, but it is unlikely to cause microcracks.

It is well accepted that the erosion rate is a function of the impact angle of particle impact velocities [1,4,6,7]. With the erosion mechanisms established, the erosion subject to the failures caused by the two components of the impact velocity can conclude that vertical plastic deformation may not produce material removal directly but it creates the potential volume that can be removed by a horizontal deformation. For oblique impacts, material removal depends upon a good depth of vertical plastic deformation with a horizontal movement. For normal impacts, the material loss can only be subject to the microcracking and fracture of the material. So, it would be improper to characterise the erosion of a material by involving the use of impact angles with other material properties at the same time, because the energy transmission has significant changes with the angles.

Different types of materials have different erosive responses to the impact angles. The erosion rate of ductile materials has a maximum value at the angle between 20° and 30° and a smaller value at 90°, whereas the erosion rate of brittle materials increases with impact angles and reaches the maximum value at or near 90° [69]. It has been observed that erosions of elastomers and plastics are generally similar to the erosion behaviour of ductile materials [70,71]. The erosive response will be subject to the erosive failure mechanisms that have been discussed. For previous studies, it can be concluded that vertical plastic deformation is essential for any erosive wear but lateral failure along the surface is the only reason for the level of erosive wear.

### 3.3. Particle Size and Shape

From Equations (1) and (2), it is recognised that the erosion damage is proportional to the kinetic energy carried by the impact solids. Large particle carries out more work so more material removal is expected at one impact. Some studies proposed a power law relationship between erosive wear and particle size [66,72]. However, Finnie [64] concluded that the volume removed by a given mass of abrasive grains was independent of particle size for particles larger than about 100 μm [73]. In recent studies, the effect of particle size on erosion was described by a master curve, which suggested that the cut-off size was about 150 μm [72]. This has been explained by the decrease in the probability of the number of particles impacting the target surface, which has the same weighting as that with the increase in their size. Consequently, for multiple impacts, the erosion can be an accumulated failure in shear planes under compression. This hypothesis means that any larger particles will not generate a large energy dissipated in the targets, which attributes the energy required to a specific failure mechanism [72].

On the other hand, smaller particles carry out smaller works with less kinetic energy as proportionally to particle size. A previous work suggested that the erosion decreased proportionally when the particle size was decreased [74]. When the particle size gets too small, it becomes hard to generate any plastic deformation required for material removal unless the material failure is caused by other mechanisms such as localised melting due to the heat generated [75]. The importance of particle shape on erosion was realised in early times [64,73]. The variation in particle shape can lead to significant changes in the erosion rate due to different mechanisms of material removal. Salik et al. [76] reported a change in erosion value by an order of magnitude due to the variation in particle shape. It was found that the angular particles were much more aggressive and caused higher erosion than the rounded or spherical particles in a study where 1018 steel was impacted at the velocity of 20 and 60 m/s by spherical glass beads and angular SiC [77]. However, it is hard to differentiate the magnitude of the influences between particle size and shape.

### 3.4. Loading Stress and Contact Areas at Impact

Recently, the effect of particle shape has been treated as shear energy produced in a unit area due to its major role in the material removal process [78]. The attention becomes more focus on the contacts between particles and the surface due to particle shapes and the dynamics of the particles such as momentums and rotations of particles. This reveals that the consequent deformations of surface materials and material removals are subject to the loading stress created in the contact only, which depends on the level of kinetic energy carried by the particles and the contact at the impact. The individual parameters of particle dynamics have been studied as mentioned above, but the loading stress created in contacts has been little investigated.

**Figure 3:**
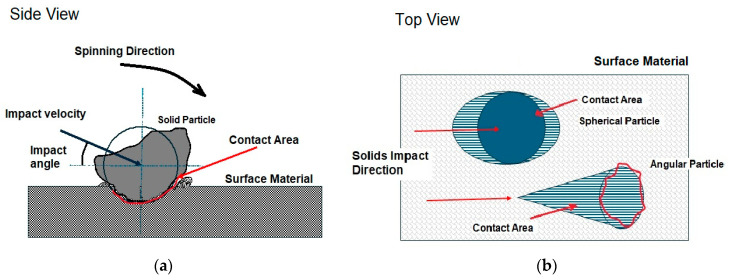
Sketch of the oblique impact of a solid particle: (**a**) impact at the contact; (**b**) the contact areas of a spherical or an angular particle.

As shown in Figure 3, the erosion damage is caused by an accumulated material removal of individual damages. The size of removed materials is subject to vertical plastic deformation (not resulting in the material removal directly but is essential for any material failures) and horizontal failures including cuttings and fractures. The vertical plastic deformation depends on the loading stress created by the solid particles, which is the ratio of the contact force to the contact area. The contact force (*F_c_*) is subject to the momentum change in the particle and the contact time (Δ*S*) as shown in Equation (3), which can be described by the change in particle velocities if the particle is rigid.

(3) $$F_{c}=\frac{{\Delta U}_{m}}{\Delta S}=\frac{U_{ma}-U_{mb}}{\Delta S}=\frac{m(v_{a}-v_{b})}{\Delta S}$$

Consequently, the loading stress (*σ_c_*) at the contact can be expressed in Equation (4), where the contact area (*A_c_*) is a function of particle size and shape as shown in Figure 3. Also, the contact area is a time-dependent function during the impact (Figure 3b), in which the contact area increases with the deformation increased, while the particle velocity decreases from the beginning of the contact until reaches zero.

(4) $$\sigma_{c}=\frac{F_{c}}{A_{c}}=\frac{m(v_{a}-v_{b})}{\Delta S\cdot A_{c}}$$

Compared to the loading stress created by the solids to the compression property of the surface material, the erosive failure under compression subjects to the resistance of plastic deformation or fracture toughness of the material. However, this is only the essential condition of an erosive failure for a material, but it is not an indication of how quick the erosive wear will be for the material.

### 3.5. Particle Rotation and Spinning

The influence of particle rotation or spinning on erosion has been studied for many years. Hutchings [4] introduced different types of cutting mechanisms based on particle rotations during the impact. In Figure 3a, it can be seen that the centre of impact velocity is often offset to the contact point of impact, resulting in a forward rotation of the particle, which creates raised lips around the craters. It frequently happens in oblique impacts whatever the particle size and shape are. If the particles have backward spinning before the impact, it can create extra cuttings. The particle rotation that happens in the impacts has relatively less effect on erosion, but it could be significant if the particles are spinning before the impacts at a high speed [79].

For a practical erosion problem, it is hard to justify the influences of particle rotation during the impact, although the particle rotation velocity before the impact is able to be measured [80]. In the energy transmission theory, it was estimated that about 1–10% of the kinetic energy of the particles was taken away by the particle rebound [32] and less than 1% of the lost kinetic energy due to particle rotation [68]. The influences of particle spinning may be not as significant as the other effects of particle dynamics. However, if the particles have significant rotation before impact, the effect of particle spinning on erosion cannot be ignored.

## 4. Influences of Material Properties

Since erosion is a kind of material failure due to fatigue, fracture, shearing, deformations, and localised melting, despite the influences of particle impact dynamics, more attention has been paid to the influences of material properties such as hardness [73,81], Young’s modulus [82], fracture toughness [40,65,83], strain rate [84], resilience [85], heat conductivity [86], melting point [58], etc. On the other hand, the material properties of solid particles such as hardness and toughness strength (resistance to fragmentation) were also considered and studied [87], which shows that the aggressiveness of solids also has an influence.

The influences of material properties on erosion have been studied in detail according to different types of materials [6]. For ductile metals, Finnie [1] used plastic flow stress created in the material to predict that the volume was removed by erosion if particles acted at cutting wear but failed at 90° impacts. Bitter [2,3] used plastic deformation for erosion prediction as a result of work hardening and cracks. With extensive studies of other materials, more material properties were considered in the erosive wear models, i.e., Young’s modulus and fracture toughness. However, more attempts to correlate the erosive wear and the material properties were made and more difficulties were found [7,87]. Because these material properties were designed for quasi-static mechanical analysis such as hardness, there was a challenge to use the material properties in the analysis as erosion was due to dynamic failures. In the erosion process, the microstructure of the surface materials and the material properties are not constant [88] and can change with time due to multiple impacts. However, these material properties are still key in the evaluation of erosion resistance for a material [89]. Some important material properties are discussed here.

### 4.1. Hardness

The effect of material hardness on erosion was probably studied first by Goodwin et al. [73], who reported that harder materials experienced less erosive damage for the pure materials. In their studies, the higher hardness of heat-treated steel had little effect. However, a later study on erosion between steel and aluminium alloy showed something different: the increase in erosion rate was directly proportional to the bulk hardness [90].

In 1977, Sheldon [82] published a study on erosive wear of six pure metals, which demonstrated a logarithmic relationship between erosion rates and Vickers hardness. Later, similar results on the influence of hardness were reported [59,91]. Divakar [92] presented a study of the effect of surface hardness on the erosion of austenitic stainless steel by cold rolling, which showed a higher erosion resistance with an increase in hardness as a result of the high compressive stresses of the target surface that was treated by cold rolling and a compound layer produced by nitriding. It contradicted what Finnie presented [87] that the erosion resistance of pure metals had a better correlation to the work-hardened hardness than steel. In recent works [93], it has been shown that the improved mechanical and structural properties of the component materials’ surface by the application of hard materials such as tungsten carbide on the surface of the component materials (generally for steel and alloys) strengthens erosion resistance properties [94,95,96].

There is a common sense that harder material provides a better resistance to material erosion, but it is hard to use the hardness in predictions of erosion although a number of erosion models included it as a parameter [6]. For example, in the 1990s, Goretta [97] had the same conclusion that higher hardness of a material improved the erosion resistance for nickel and stainless steel if it retained sufficient ductility, although, in the early 1980s, Sundararajan [22,81] concluded that neither heat treatment nor cold working of metals had any significant effects on erosion resistances. As the hardness of a material is defined as its ability to withstand localised permanent deformation, a high hardness means a high resistance to deformation due to other actions including erosions under compressive stress. Although hardness is normally measured in quasi-static loading rather than dynamic loading, it is still able to show the ability to restrict plastic deformation. In the erosion process, if the failure mechanism is plastic deformation by loading stress only, the erosion would have a better correlation with the hardness of materials such as pure metals.

### 4.2. Young’s Modulus and Yield Strength

For erosion, using the Saint-Venant theory of elasticity [98] and the Hertz equation the maximum force at the contact of colliding bodies and the plastic deformation could be calculated theoretically [2,3]. With the development of fracture mechanics by considering elastic–plastic deformation, prediction of depth of damage and strength of degradation was used for brittle materials [55,99]. However, Sheldon [82] mentioned that Young’s modulus and other material properties related to bond strength or annealed hardness would not provide a good correlation to the erosion of metals such as steel. Nevertheless, elastic properties such as Young’s modulus and yield strength are still important in many elastic–plastic deformation theories of erosions [37,55,100,101].

Young’s modulus is a measure of the stiffness of an elastic material and defined as the maximum ratio taken of stress to strain before plastic deformation. For erosive failure of materials, elastic deformation is an essential stage before any plastic deformation or fracture failures happen. During an impact, the elastic deformation absorbs part of the kinetic energy and dissipates through an elastic wave without causing damage. Higher Young’s modulus and yield strength mean more kinetic energy of the impacts is absorbed and gives better erosive resistance to the material at the same impact condition. These material characteristics may be not suitable for correlating the erosive rates directly but could be used for an indication of erosion resistance of a material to an impact deformation or an explanation of erosive mechanisms. These elastic properties of materials can be important, especially for elastomers [102,103].

### 4.3. Fracture Toughness

Bitter [2,3] considered the erosion as a low-cycle (high strain) fatigue process, but the theory did not agree with the experiments very well. Hence, Hutchings [4] suggested that cuttings, adiabatic shear failure, and high cycle fatigue could work for erosion mechanisms of ductile materials. Fatigue cannot explain the erosion of brittle materials directly, as the failure is likely due to the fracture cracking of material grits [8,104,105], which is defined as the separation of a material into two or more pieces due to stress exceeding its strength, below its melting point, leading to a loss of structural integrity. Fatigue failure can be one of the fracture failures under an accumulation of micro-fractures in the deformed part of erosion damage.

Fracture failure is another important erosion mechanism and is different to plastic deformation, so fracture toughness has been brought into a number of erosion theories for brittle materials [19,40,54]. Because fracture toughness is the resistance of brittle materials to the propagation of flaws or cracking under applied stress, it has been used to describe the brittleness of material plasticity and energy absorbed by crack propagation in a number of peoples’ work [7,100,106,107] as a useful indicative factor to the material’s ability absorbing impact energy without fracture. However, the fracture toughness is hardly found in the models for ductile materials. Instead, hardness seems much more comprehensively used in theoretical models for both brittle and ductile materials [7].

Shipway’s study showed that fracture toughness was a strong factor in the erosion of brittle target materials [108]. Recently, fracture toughness has been used for calculating stress-intensity or critical stress in the theories of crack or microcrack propagation incorporating indentation tests [109,110].

### 4.4. Microstructure and Thermal Properties

Since erosion of a material is caused by a number of failure mechanisms or a combination of the failure mechanisms, erosion resistance of a material is never subject to any individual material mechanical properties. The influence of material microstructures on the erosive behaviour of metals was recognised many years ago [38,111]. Levy [89] studied the erosion of steel as a function of microstructure and concluded that the microstructure played an important role in the erosion of ductile alloys, as ductile ferrite and brittle pearlite behaved differently. However, some research showed that the microstructure of alloy steel and high-speed steel did not affect their erosion resistance positively [112]. Later research confirmed that the erosion response of pearlite and ferrite were different depending upon particle impact location and the orientation of the microstructure relative to the impinging particle [113]. Compared to the solid particles, the affected areas by the impacts are comparative to the microstructures rather than the bulk material. If a material consists of a complex structure such as composite materials, different microstructures with different erosion responses must be significant.

The studies [16,114] show that the microstructure of materials may not have a direct impact on erosion, but material properties of different phases in the microstructure give different erosion resistances under the impacts. The hard phase does provide a good erosion resistance to the ductile matrix. Comparing erodent size to the dimensions of the microstructure, transition or bonding strength between the phases can be important in the erosion of a composite material such as steel. The grain size of harder phases may not take responsibility as expected, but the deformation of the ductile transition phase can be reduced by the harder phases [90,112].

It is noticed that the heat generated in the impact can alert the mechanical properties of the microstructure, which influences the erosion resistance of the material [115]. Therefore, using heat treatment to improve erosion resistance hardly works for a composite material [92,116]. The influences of localised melting or microstructural change due to localised heat on erosion have been investigated since the 1970s [4,79,117,118]. Söderberg [119] showed some photographic evidence of local high temperature generation, which demonstrated that localised melting could be a reason for material removal. However, it is not easy to measure the local temperature rise during erosion practically. In 1968, Neilson [120] estimated a temperature rise of 300 °F during impact, although evidence of melting was not observed. However, Brown [75] suggested only an approximate 100 °C temperature rise due to localised heat, which was not significant in changes in microstructures and properties. Later, Hutchings [121] concluded that no surface softening occurred, but there was evidence of recrystallisation in the erosion debris and close to the surface regions for the erosion of ductile metals by the thermal effects.

The thermal effect on erosion has been little studied. With the progress of numerical simulations, the theoretical estimation of an instant surface temperature produced by a single impact was up to 1000 °C and stabilised at 60–300 °C in the impact of stainless steel [122]. Since localised plastic deformation induced by the impact of particles and the adiabatic conditions prevailing at high strain rates may produce high temperatures, the hardness and fracture toughness of the target must be influenced by the thermal field and thus the erosion resistance of the parts [123]. Therefore, thermal properties including thermal conductivity and temperature resistance of the target materials can be important parameters for the erosion process, which are not only altering microstructure but also influencing localised melting and consequent erosive resistance.

### 4.5. Ductile Materials

**Figure 4:**
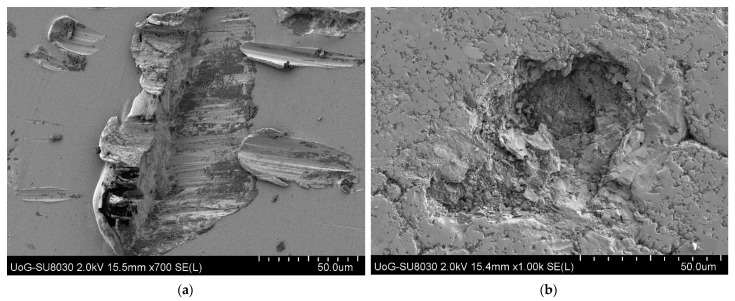
Eroded material surface: (**a**) Ductile material (steel), mild steel (EN42) (sands impact at 40 m/s and 45°). (**b**) Brittle material (SiC), silicon carbide (SiC) (sands impact at 40 m/s and 90°).

Erosion of ductile materials has been recognised as the removal of materials caused by cutting or ploughing at low angles [4] or continuous plastic flow [90,124]. Many theories developed for ductile material erosion are based on plastic deformations, in which craters are formed and a major part of displaced volume forms a raised ridge around the circumference of the crater and the lips (see Figure 4a of our previous work) [8,125].

In the erosion of ductile materials, ductility is thought as an important material property, which is the ability of a material to take plastic deformation before fracture. Plastic deformation is an essential stage before material removal failure under applied stress, as opposed to elastic deformation which is reversible upon removing the applied stress. Ductility has been used for evaluating erosion of ductile materials by many researchers in varied theoretical models and their study confirmed that higher ductility resulted in lower erosion rates [126,127,128]. However, ductility is less common in theoretical models compared to other material properties such as hardness and fracture toughness [7].

It has been recognised that erosion of ductile material happens in a two-stage process, in which surface material is extruded first and then gets forged in subsequent impacts, so material removal is a kind of extrusion-forging [127,129]. Initially, large craters are formed, hence, larger platelets, but with progressing wear, the craters and platelets overlap and lead to the formation of smaller craters and platelets (see Figure 4a). Material melting during impacts could happen [121], which may lead to changes in material properties, hence a different erosion rate.

Levy [127] claimed that the steady erosion rates of three different heat-treated materials related directly to their ductility, but many researchers did not believe that because of the localised heat influence during the impacts [76,81,130]. Levy [127] accepted that the primary erosion mechanism of ductile materials was high strain formation, which the stain rate might be far higher than that obtained in common mechanical property tests. Hence, the relationship between the ductility and the erosion of ductile materials is obvious [131], but it is hard to correlate directly.

### 4.6. Brittle Materials

Compared to ductile materials, the erosion of brittle materials is different and caused by the propagation and intersection of cracks under solid impacts. Brittle materials only take little plastic deformation and form microcracks when the loading stress exceeds the limit of toughness (see Figure 4b). Finnie was probably the first person to suggest that erosion could be classified into two categories according to the responses of the damaged materials as the result of two major erosive mechanisms; plastic deformation and cracking fracture [132]. Compared to ductile materials where the maximum erosion rate occurs at the impact angle of 20–30°, the brittle materials have the maximum erosion at the impact angle of 90°. However, the difference in erosion behaviour between ductile and brittle materials sometimes is not that clear. In 1995, Finnie summarised the erosion mechanisms of ductile and brittle materials and discussed the transition in erosion behaviour of a normal brittle material to ductile behaviour as a result of a reduction in erodent size, which revealed an interesting phenomenon of erosive behaviour transformation in-between two typical erosion mechanisms [87]. Brittle behaviour seems to happen when the grain size in the material is comparable to the grit size of the particles.

In the 1960s, Sheldon and Finnie [133] developed an erosion model of brittle materials by fracture mechanics based on cracking under indentation. Later, Evans et al. [54] showed some remarkable progress in the approach followed involved elastic–plastic behaviours by assuming the lateral crack size was proportional to the radical crack size and only taking the depth of the lateral cracks. Recent studies suggest that the erosion of brittle materials could be classified into two categories depending on the morphology of the indenter or erodent such as fracture and fatigue-crack propagation [134,135]. For blunt particles like spheres, cone-shaped cracks or Hertzian cracks were formed, and at higher impact velocities, radial cracks which originated were typically formed due to sharp indentation [67].

With more experimental results obtained, some conflicting observations were discovered [136,137]. Ritter summarised the studies of the role of microstructure and the second phase in the erosion of ceramics [138]. According to the indentation theory [45,54], the normal impact of a solid particle on a brittle material can generate a plastic zone beneath the particle–material contact area. Therefore, fracture toughness and cracking theory do seem not to solve the problem completely. Tarodiya et al. [16] summarised that the erosion of brittle materials may involve grain boundary cracking, grain ejection, ploughing, and chipping out of lateral cracks caused by the impact of particles, as well as the plastic deformation of the surface materials at the same time. For erosion of brittle materials, Melentiev [6] pointed out that most efforts made were developing a suitable crack system and correlating its parameters to the known material properties in the frame of fracture mechanics. However, these theories yielded some critical drawbacks during analysis, as the material properties and the impact conditions are not independent.

### 4.7. Elastomeric Materials

Elastomeric materials are usually a group of polymers with special characteristics such as high elasticity and viscoelasticity, which regain shape when the stress applied is removed and the glass transition temperature is far below room temperature. Elastomers are poorly crystallised but more crystalline as they are stretched. Sometimes, elastomers show excellent erosion resistance, which makes them great for use as protective coatings and liners in large-scale industrial applications [139,140].

The erosion mechanisms of elastomers are subject to the material properties of erodent solids and impact conditions [16]. Previous studies suggested that the erosion of the elastomers was due to fatigue cracks on the surface by tensile, compressive, and shear stresses caused by each particle impact and the removal of chunks of a few microns due to the intersection and extension of the formed cracks [103,141]. The other minor mechanisms of erosion for elastomers can be the formation of ridges due to plastic deformation perpendicular to the direction of particle impacts and their removals during subsequent particle impacts [142]. Cutting and ploughing of the surface by sharp angular particles are also thought as an important erosion mechanism of elastomers [143].

High elasticity or high viscoelasticity is the special characteristic of elastomers, which influences the erosion behaviours of this type of material strongly [104]. As discussed, elastic deformation is an essential stage before the material suffers from any failure removal. The elastomer materials can absorb a large proportion of the kinetic energy during the impact so less energy results in the failure removal of materials such as tearing or cracking in deformed regions [139,144]. The influence of elasticity or viscoelasticity on the erosion of elastomers was investigated recently, which showed a good correlation between the erosive mass loss and the loss modulus [145] and the viscoelastic behaviour over a wide range of strain rates [146].

Compared to metals such as steel, the hardness and Young’s modulus of the elastomeric materials are relatively low, which can be thousands of times lower than steel. However, Xie’s study [139] showed that the erosion rate of steel was much higher than that of elastomers. The plasticity index, defined as hardness over Young’s modulus, was claimed to be proportional to the maximum elastic strain beyond the plastic deformation occurred, which the resistance of elastomers to take plastic deformation is much higher than steel [147]. However, low surface hardness means that it is not good to take any resistance of loading stress which causes tearing or cutting failure when the solids are sharp or angular. A recent study showed that the highest erosive wear of elastomeric coatings was recorded at the impact angle of 45°, which indicated that the material failure was subjected to a shearing mechanism [148].

### 4.8. Composite Materials

Unlike other common materials, composites in which two or more distinct, structurally complementary materials are composed to produce structural or functional material properties [149]. Erosion of a composite material can be different to the single-phased materials and is much more complicated. The erosion resistance of a composite is subjected to the individual phases and the bonding strength between the phases. Because erosive responses to different phases or bonding strength can be significantly different, the erosion of a composite will be subject to the weakest parts in terms of components, structures and reinforcement fillers. Commonly, the composites are classified based on matrix materials such as polymer, metal and ceramic, which are reinforced with various materials such as fibres and particulate fillers [17].

A review [150] on the erosion of polymer composites revealed that the incorporation of both fibre and filler particles in matrices often referred to as hybrid composites can provide improved erosion resistance. A study indicated that the interface between matrix and fibre/filler reinforcement bonding played a key role in affecting erosion rate [151]. As an example, the erosion mechanisms of composite materials are not different to those of other common materials studied previously. However, the interface between the phases has more obvious influences on the erosion behaviour of the composites because of the structural differences in and between the reinforcement, fillers, and matrix materials [52]. Therefore, the overall erosion performance of a composite is subject to not only the erosion resistances of the individual components but also the bonding strength at the reinforcement and matrix interface. The structures or even the microstructures of the composites can be important to the erosion resistance.

Compared to the distinct materials, composites used to have a larger material structure compared to erodent grain size. This can be important because the solid particles may strike on different material structures and it results in different material removals at the macro level under multiple impacts. The reinforcement of a composite proposes to produce a strong bonding strength and hold the matrix materials together. However, in most cases, complex architecture (compared to monolithic counterparts) could contribute to irregular erosions due to the failures of the reinforcements [152].

## 5. Thinking of Erosive Wear More Fundamentally

It can be concluded that any intention for an accurate prediction of material erosions is not feasible by any existing theories because of the complexity of the process and the number of parameters involved in erosion [16]. Efforts of numerical simulations were made but it seems facing the same barriers as the theories. The trustworthy evaluation of erosion seems to be the empirical tests only, whereas the applicability of the theoretical equations for prediction of erosion is limited to certain types of surface materials such as hard surfaces.

However, the current recognition of erosion and its prediction might be incomplete, with the existing theories used to treat a process as a material property and aim to use all variables involved in the process for an accurate prediction. With the studies, it can be realised that the erosion of a material is a progressive material removal due to impacts of aggressive particulate particles, which the process involves three aspects, solid particles, surface materials and impact dynamics and is uncontrollable. The material removal from a surface is subject to two conditions: erosion resistance of the surface material and aggressiveness of the particulate particle impacts if the impact dynamics is fixed constantly such as particle velocity and impact angle. The impact conditions can be independent of the erosion resistance and aggressiveness. Conversely, the erosion resistance and the aggressiveness may depend on the impact dynamics such as impact angles.

### 5.1. Erosion Resistance of Materials

Erosion resistance of materials is thought of as surface failure strength when the material starts to lose functional strength between the affected part and the parts connected and consequently, the affected part is removed from the original location. This is the only reason why the erosion rate of a material is defined as the mass loss or volume removed by a unit mass of particles impacted.

There is a contradictory point between the erosion rate and the erosion resistance of a material. Since the 1950s, erosion has been seriously considered in quantity forms in terms of solid particle dynamics, material properties of the surface and the solids, and other influential factors such as heat capacity and temperature rise. Tarodiya [16] gave a good summary of theoretical equations used for erosion prediction and concluded that two methods were commonly used to derive the erosion equations based on either solving the equation of particle motion while impacting the target surface or applying the energy conservation principle during the interaction. In solving the equations, the removal of material swept by the particle is converted to a non-dimensional form as the erosion rate. Rather than giving a definite form, a general formula used to be as follows:

(5) $$Erosion\;rate\;\left(\varepsilon \right)≅\varepsilon_{c}+\varepsilon_{d}≅\left(K_{c}+K_{d}\right){\left(v_{p}\right)}^{n}$$

where *ε_c_* and *ε_d_* are the erosion rates according to the erosion mechanisms of cutting and deformation. *K_c_* and *K_d_* are the constant coefficients as a function of impact angle, material properties of particles, and target materials.

**Figure 5:**
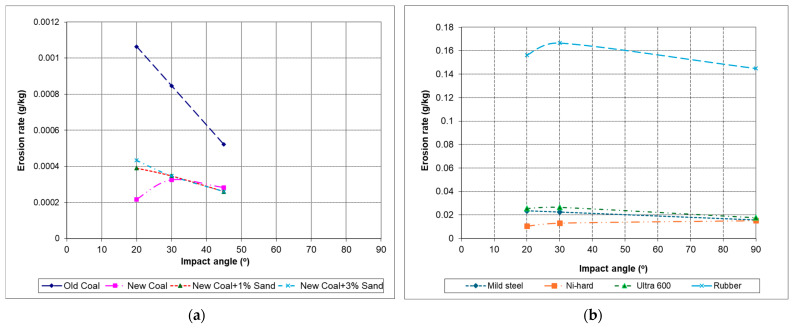
Erosion rates of (**a**) mild steel impacted by erodent (coal < 1.18 mm) at 20 m/s; (**b**) different surface materials including mild steel impacted by the same erodent (Chrome < 0.7 mm) at 20 m/s.

The problem of using erosion rate for prediction is that the uncontrollable variables such as particle sizes, which is impossible to synthesise erosion equations from the existing erosion mechanisms. In other words, the erosion mechanisms involve many different types, but in theoretical models, the erosion rate was calculated by the parameters related to the erosion mechanisms for establishing correlations. Therefore, more than 100 variables and constants were used in 30 plus equations in the calculation [7]. Erosion rate is not a standard material property, which is subject to many conditions in an erosion process. An example of a previous study at the Wolfson Centre on the erosion of steel by sand-contaminated coals and different types of surface materials by Chrome particles is shown in Figure 5. It shows that the same surface material (mild steel, EN42) has different erosion rates by different erodent particles (coals) in Figure 5a and different surface materials by the same erodent particles (Chrome) in Figure 5b at the same impact velocity.

The erosion resistance of a material could be constant if it is taken as a parameter of material properties. Erodent particles can influence the erosion rate of surface materials but should not change the erosion resistance of the materials unless the structure or microstructure of the materials is being altered during the process such as temperature influences. Decoupling particle aggressiveness and impact dynamics from the evaluation of erosion resistance for a material could be more appropriate by defining the erosion resistance as a property of the material, which can be defined as a parameter of specific toughness multiplied by the permanent deformation distance generated by the impact.

It can be concluded that the erosion rate given in Equation (5) will never be constant, and the volume or the mass removed by the unit mass of solids will be subject to the aggressiveness of particulates and the impact dynamics conditions. If the impact conditions are fixed, the erosion resistance of a material can be constant, which will only depend on its mechanical properties under dynamic loadings.

### 5.2. Aggressiveness of Particulate Particles

For erosion of a material, there is an obvious link between the erosion rate and solid particles and their impacts. Consequently, influences of particle properties and impact dynamics seem essential for the prediction of erosion and have been used in all the theories [66,153,154,155]. However, it is questionable whether it is adequate to involve the particulates and the impact dynamics or not when the erosion of a surface material is evaluated.

It is undoubted that different solid particles contribute different erosion rates of surface material in the circumstance of identical impact dynamics. Even with the same solids with different particle sizes and shapes, the erosion rate will be significantly different [109]. If erosion of a material is justified by the erosion rate, the quantity of erosion will seriously depend on the solids interacting with the surface and never have a repeatable value.

To decouple the influences of particles and the impact dynamics from the evaluation, it could be essential to use the solids with the same aggressiveness, so the material removal will not be influenced by the ability of solid particulates. The aggressiveness of the solid particles can be defined as the maximum loading stress that the solid particles can produce before degradation as follows.

(6) $$Maximum\;loading\;stress\;\left(\varepsilon_{m}\right)=\frac{m\left(v_{i}-v_{r}\right)}{A_{c}\cdot \Delta S}≅\frac{mv_{i}}{A_{c}\cdot \Delta S}\;(Pa)$$

where *m* is the mass of solids, *v_i_* is the impact velocity, *v_r_* is the rebounded velocity which can be zero approximately if the particle is smashed at the impact, *A_c_* is the contact area, and Δ*S* is the contact time of the impact. The contact area is subject to the size and the shape of particles and the contact time will be subject to the material properties of the solids and the surface. In Equation (6), the mass and the contact area can be simplified if the solid is a spherical particle as in Equation (7).

(7) $$Maximum\;loading\;stress\;\left(\varepsilon_{m}\right)≅\frac{mv_{i}}{A_{c}\cdot \Delta S}≅\frac{4R\rho_{d}}{3\phi }\left(\frac{v_{i}}{∆S}\right)\;(Pa)$$

where *R* is the equivalent radius of solid particles, *ρ_d_* is the solid density of particles, and *ϕ* is the ratio of the contact area to the projected area of the particle.

**Figure 6:**
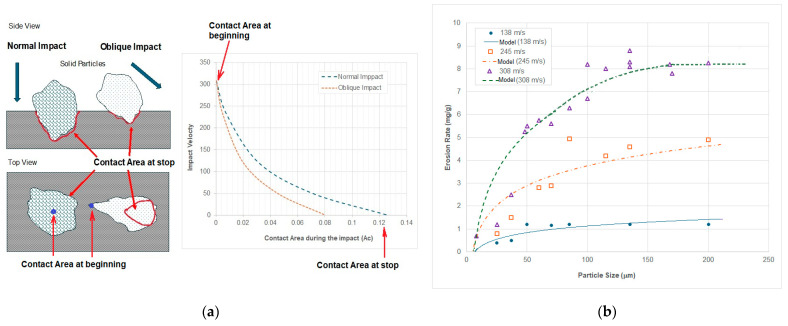
Influence of particles on erosion: (**a**) contact areas of a blunt particle; (**b**) erosion rates of an 11% chromium steel for the normal impact of quartz particles (reproduced using the results [73]).

Obviously, the aggressiveness of solid particulates will be subject to the hardness and fracture toughness of the solid particles. If the loading stress created at the contact point is bigger than the maximum loading stress that the particles can produce, the solid particles will be smashed and lose their aggressiveness. As shown in Figure 6a, it illuminates the contact areas that the impact particle may produce during a normal impact or an oblique impact, which will be slightly different. However, the initial contact areas at the beginning of impacts are similar whatever the impact angle is. The contact area increases while the impingement is ongoing until the maximum depth of the crater is produced. With an increased particle size, the erosion rate produced becomes a constant level approximately because of the increased aggressiveness of particles compensated with the reduced number of particles as shown in Figure 6b.

### 5.3. Assessment of Erosion and Questions Remained

The previous studies on the erosion of different types of materials show that assessments using erosion rates are not reliable, which seriously depends on test conditions. The major difficulty of the assessments is the number of uncontrollable variables involved in erosion regarding material properties and impact dynamics. Measurement of these variables is not repeatable under different test conditions. Additionally, erosion is a progressive process, where the material properties may have a continuous change in the process while the failure is accumulating such as fatigue fractures. Therefore, the best assessment of erosion is limited to experimental measurements only, as any theoretical prediction cannot track the historical changes in the material properties and the particles except numerical simulations [6].

The previous studies had recognised that the impact dynamics, the material properties of both particles and surface materials, and even the surface conditions all had strong influences on the results of erosion assessments [16,94,156]. In summary, there are still many unsolved questions remaining in the topic, which really set barriers to any progress on assessment and prediction of erosive failures of materials. With the discussions in this paper, some major unsolved questions remain could be as follows:

*The first question is how to simulate the progressive changes in material properties in multiple particle impacts until material failure or removal happens*. Material erosion is caused by cumulative impacts of solid particles, which can have huge variations in the material properties from one impact to the others and are apparently dependent on the impact conditions. For single particle impact, the material properties and the impact conditions could be identified easily, i.e., particle size and shape, impact velocity and angle, energy transmitted during the impact, material deformation under the impact, etc. However, with multiple-particle impacts, these are never easily constant over the period of the erosion. Most of them are random with a wide range and can have a progressive change. Taking a value of the material properties in the calculation of erosion seems reasonable but not realistic. Monitoring the progressive changes in material properties in erosion could be essential but cannot be achieved without using numerical simulations.

*Secondly, how to decouple the influences of solids from the erosion prediction could be an essential question*. If it is achieved, it may reduce the complexity of erosion prediction and evaluation significantly. In the existing theories, erosion of materials used to be quantified by mass loss or volume removed under unit solid particles impacted (kg/kg or m^3^/kg). The erosion rate of a material is a common material characteristic, but it seriously depends on the solids used in the tests and the impact dynamics conditions applied. Therefore, the erosion rate of a material is never universal. Using the erosion rate of a material seems not fair for any comparison between materials unless the solid particles and the impact conditions are identical. Even for a prediction of an erosion process, the erosion rate is not reliable because of the inconsistency of the solids and the impact dynamics. So, for an accurate prediction or assessment of erosion, decoupling the influences of solids can be essential so erosion could be evaluated only by the same aggressiveness of solid particles.

*Another question is what a proper way could be to assess the erosion of a material*. Regarding the erosion discussed in this paper, it seems not feasible to establish a standardised approach for the evaluation of erosion, which would be more suitable for industrial applications. Because of the complexity of erosion, it seems that more people who are working on this topic have lost their interest in any attempts at new theories or novel approaches for the prediction of erosion problems instead of using numerical simulations. Assessment of erosion is not particularly easy by any existing theory or experimental test approach, although all the theories present some useful insights into the nature of erosion as concluded recently [6]. As discussed, any kind of erosion leads to a material functional failure by material removals. The material functional failure does not mean the material removal, but the removal of materials must follow the material failures caused by solid particle impacts. The failure and the removal of materials are the results of multi-millions of particle impacts and an accumulation of contacts and interactions, which makes any mathematical calculation or theoretical prediction impossible.

### 5.4. Limitations of the Existing Theories for Practical Challenges

A number of reviews on mechanisms, theories, and predictive models for erosion of different types of materials have been conducted [6,16,137,150,157]. With the reviews, it can be concluded that the existing theories yield some critical drawbacks during analysis and rigorously speaking, all the theories could not provide a realistic prediction of an erosion process [6]. The theories of erosion mechanisms can provide some useful insight into the nature and a reasonable explanation of erosion, but their mathematical forms and applicability are all subject to material types and test conditions specified. These existing theories are limited by a number of restrictions on tracking any changes in material properties in erosion due to the impacts, material failure history, and material removals due to the accumulated material failures.

The biggest limitation of the existing theories is the numerous uncontrollable variables used in the models. Over the years, many erosion equations have been reported by the investigators that would help the engineers to obtain a quick answer without a comprehensive measurement of erosion [6]. In the equations, more than 100 variables, constant coefficients, or parameters have been used based on material properties, erosion mechanisms, and impact dynamics. To determine the erosion rate of a material, these variables and constant coefficients need to be quantified experimentally, and the model developed will be limited to the conditions established very restrictedly. Otherwise, the model does not provide a sensible answer if the variables are different to what has been established. Many of the variables seem not controllable in practice.

The second limitation is that the existing theories cannot trace the historical changes in material properties in an erosion process, which is caused by the complexity of the erosion mechanisms due to different microstructures and structural transitions in erosion [67,149]. In the existing theories, the erosion rate was normally extracted from the indented volume by a single particle, which represents an average loss of surface material under certain impact conditions. All the theories reviewed by Melentiev [6] and the empirical erosion models reviewed by Tarodiya [16] show that the erosion prediction must correlate to the types of surface materials, which the erosion can also be influenced by the difference in dominant erosion mechanisms, erodent properties and behaviour, and the impact dynamics. The existing theories used conventional material properties for the prediction of dynamic progress, which results in a loss end due to a progressive change in these material properties in erosion. Therefore, it was concluded that the applicability of the theoretical equations was mostly limited to the type of surface materials considered and restricted to certain conditions tested [16].

For practical applications, any theoretical prediction of material working life under erosion is limited by the number of variables involved and the accuracy required. In practice, if erodent solids and impact conditions are not representable by any preselected materials or any controllable matters such as particle size and shape distributions, the prediction will be wrong. Also, the progressive change in material properties in erosion leads to another barrier for practical applications, which is even not solved in numerical simulations. In summary, any existing theories or numerical simulations are not realistic for practical predictions at present except for empirical measurements.

### 5.5. Possible Solutions in Future

Over the years, a lot of efforts on studies of erosion have been made. It is concluded that prediction of an erosion process by material properties associated with impact dynamics seems impossible. This conclusion extremely limits practical applications of any existing theories for the prediction of erosion, selection of erosion resistance materials, or a better solution for any erosion problems. The challenge comes from the variations in solid particles and the impact dynamics associated with them. Multiple impacts resulting from a large number of particles would create an unstable erosion rate over the process, which means that the material removal would be affected by the variations significantly, including material properties of the solid particles such as size, shape, and density, and the impact conditions such the contact area and the loading stress [158].

Therefore, for practical applications, it is suggested to isolate the influences of erodent properties and operating conditions from an erosion evaluation of surface materials if it is possible. It is known that the erosion of a surface material will be subject to the loading stress created by a solid particle and its lateral component. If the erodent properties and the operating conditions are not constant, the material removal by the solids at the conditions never has a constant rate so the erosion rates of the materials can have huge variations. A standardised approach for the evaluation of erosion resistance or erosion rate will require the minimum effects of the erodent properties and operating conditions so the erosion of different surface materials can be compared at the same conditions.

The other possible solution is to create a constant level of loading stress and an identical impact direction in an erosion test by using a nominated erodent material. It is similar to what was suggested by Tarodiya [16] as a universal erosion test using a universal bench-scale test rig for erosion measurement. However, Tarodiya did not describe what the universal erosion test should be and how it could be achieved. With the discussions in this paper, the universal erosion test can be undertaken using a standardised erodent material such as quartz sand with a narrow size range. The impact conditions can be selected as oblique, i.e., 45° and normal impact at 90° with designed impact velocity. As the aggressiveness of quartz sand can be standardised, erosion due to other erodent materials can be achieved by comparing the aggressiveness of quartz sand to the erodent materials rather than carrying out extra erosion tests.

There could be another solution by defining a term of erosion resistance of a material instead of using erosion rate to define the erosion property. The problem of using erosion rate has been discussed that any erosion rate of a material depends on the erodent properties and the operating conditions. Because of the inconsistency of the erodent materials and the operation conditions, the result of the erosion rate is not realistic for the working life prediction of the materials under the same erosion conditions. Even for comparison between the materials under the same impact conditions, it is not realistic because the surface materials may suffer from different erosion mechanisms. The term erosion rate has been used for nearly 70 years and was described in terms of mass or volume loss per unit erodent mass [1]. The erosion rate involves both the surface materials and the erodent material used, and it was calculated in terms of the material properties and the impact conditions. So, it is not realistic when using the erosion rate for a material property characteristic.

The erosion resistance of a surface material could be more realistic, which can be defined as the maximum stress before failure that the material can take. If any loading stress by solid particles is bigger than the erosion resistance of a material, the material will have an erosion problem. Any erosion rate measured can still have a physical meaning for a specific erosion problem, which can be used for an evaluation of the working life of a material under certain operation conditions.

## 6. Conclusions and Remarks

The erosive wear of materials by solid particles has been extensively studied in the past and massive studies have been reported in terms of fundamentals, influential factors, experimental studies and numerical simulations for practical applications. However, due to the complexity of the phenomenon, there are still many unsolved questions remaining. The major unsolved question is whether there is a universal theory that covers the full complexity of erosion or an intrinsic material property solely responsible for erosion resistance. In this paper, it is concluded that the complexity of an erosion process is due to the conjunctions of the solid particles, the responses of surface materials under impacts, and the impact dynamics. The existing theories use many uncontrollable variables from the three aspects above for the evaluation of erosion, but there is not a universal theory or a group of dynamic equations that can describe the process precisely.

Nowadays, it seems to fail further delving into any fundamentals as the data and the results obtained already allow remarkable progress in wear reduction, precision manufacturing, surface modifications, and many other applications. However, this conclusion may be inappropriate because of the lack of understanding of the progression of erosion and its influences on erosion. The existing erosion theories only considered the erosion rate as an intrinsic material property responsible for erosion, which was extracted from an average loss of surface materials under certain impact conditions. Therefore, the complexity of the uncontrollable variables in the process cannot be solved by any existing theoretical or numerical simulations of erosion.

To solve the deadlock, it is suggested to use a distinct material property to replace the erosion rate that has been used for the last 70 years, i.e., erosion resistance. It can limit the variables from the solid particles. Instead, a term for the erosive aggressiveness of particles is suggested in the paper to isolate the influences of solid particles. Using these two terms, the evaluation of an erosion process could be simplified as the impact dynamics can be standardised by the erosive aggressiveness of particles in a test. By the way, the effects of the erodent properties and the operating conditions can be minimised.

With the thinking of erosion more fundamentally, several remarks on the erosion can be made:

1. Mechanisms of an erosion process can be complicated and may have a progressive change. The failure mechanisms in erosion can be inconsistent and a combination of a few different erosion mechanisms due to the changes in material structures or microstructures, especially for composite materials.
2. An erosion process appears to involve three aspects: solid particles, surface material, and impact dynamics, which can influence each other and make the erosion rate measured specific to the erosive conditions. From the previous studies, it concludes that vertical plastic deformation is essential for any erosive wear but lateral failure along the surface is the only reason to cause erosive wear.
3. It is hard to use erosion rate for theoretical prediction or evaluation in practice. To define an intrinsic material property for a surface material, erosion resistance seems more proper instead of the erosion rate. The erosion resistance of a material can be defined by the minimum loading stress that can cause a material removal from a surface of materials due to erosive impacts regardless of the material properties of solid particles and the impact dynamics.
4. On the other hand, to isolate the influences of solid particles, the erosive aggressiveness of particles can be used as a term to describe the influences of solid particles. Obviously, the erosive aggressiveness of solid particles links with the impact dynamics. The solid particle with a higher impact velocity must be more erosion-aggressive. So, the erosive aggressiveness of particles can be defined as the maximum loading stress that the solid particle can produce before the particles are plastic-deformed or fractured.
5. Erosion rate can still be a useful term for the evaluation of erosion under different erosion conditions using erosion test rigs. As erosion rate determination involves solid particles and impact dynamics, it is not a realistic characteristic for erosion prediction or comparison between different materials.

With the novel definitions of erosion resistance and erosive aggressiveness, further research can delve into erosion by multiple impacts with a historical change in material properties. The numerical simulations of the erosion process could benefit from the use of any AI techniques associated with further understanding of erosive wear mechanisms. For improving erosion prediction models, incorporating evolving material properties with experimental validation by using advanced imaging techniques can be essential, which will help the development of standardised erosion testing methods to ensure comparability across the studies.

## Abbreviations

The following abbreviations are used in this manuscript:

FEM	finite element methods
DEM	discrete element methods
SiC	silicon carbide
CFR	Carbon-fibre reinforced
